# Physician Assistant Utilization in Inpatient Psychiatry: A Qualitative Study

**DOI:** 10.7759/cureus.11900

**Published:** 2020-12-04

**Authors:** Shaun P Curran, Mary Boyette, Alexa Callison-Burch, Joseph Hagloch, Ryan Walsh, Catherine Van Tassell, Virginia L Valentin

**Affiliations:** 1 Family and Preventive Medicine, University of Utah Health, Salt Lake City, USA; 2 Psychiatry, University Neuropsychiatric Institute, University of Utah Health, Salt Lake City, USA

**Keywords:** inpatient psychiatry, physician assistant, workforce

## Abstract

Objective

There is a national shortage of psychiatric care providers, with approximately 1% of physician assistants (PAs) working in psychiatry. The study aimed to understand the utilization of PAs in inpatient psychiatry.

Methods

A qualitative study was performed utilizing semi-structured interviews focusing on PA autonomy, reimbursement, specialized certifications, training structure, and overall satisfaction with PAs in inpatient psychiatric care.

Results

Of the nine locations interviewed, four are currently using PAs, and five have never utilized PAs. All facilities utilizing PAs reported a decrease in physician workload with varying structures for training and billing, and required specialized certifications.

Conclusion

Most facilities surveyed do not utilize PAs and either preferred physicians or were unaware of the qualifications of PAs. Of the facilities utilizing PAs, there is wide variation in their utilization and reimbursement models; however, they reported a high level of satisfaction, reinforcing that PAs can provide high-quality care in inpatient psychiatric settings.

## Introduction

Nationally, there is a shortage of psychiatric care providers [1]. The population of practicing psychiatrists declined by 10% from 2003 to 2013 and this current shortage is predicted to keep growing as providers age out and retire [1]. At the same time, the demand for psychiatric care is only increasing with a projected shortage of 15,600 psychiatrists by 2025 [1]. Advanced practice clinicians (APCs) have been proposed as a viable solution in mitigating the shortage of providers and filling in the gaps in psychiatric healthcare [2]. Yet, the method of employing APCs in psychiatry is fragmented and inconsistent [2].

Only a small number of physician assistants (PAs) account for the psychiatric APC workforce. According to the 2018 National Commission on Certification of Physician Assistants (NCCPAs), 1.5% of certified PAs work in psychiatric care [3]. The NCCPA does not differentiate between inpatient and outpatient care, so the number of inpatient psychiatric facilities utilizing PAs is not known. The lack of PAs in psychiatry is so severe that it prompted an editorial in 2010 titled, “Where are the Psychiatric PAs?”[4].

One reason for the shortage of PAs in psychiatry may be due to a deficiency of understanding how to utilize PAs. PAs are employed in mental health care from outpatient clinics to community jails to inpatient psychiatric units [3]. Yet, there is no published literature to date on PA utilization in psychiatry including PA role, training and reimbursement models.

To strengthen the psychiatric PA workforce, two solutions have been developed: post-graduate training programs and Certification of Advanced Qualification (CAQ) in psychiatry. Advocates of post-graduate training programs promote benefits to the trainees through formal psychiatric training, benefits to the institution through the work of well-trained providers and benefits to the community through an increase in psychiatric care providers [5]. Despite the interest in this solution, the Association of Postgraduate PA Programs lists only three psychiatric fellowships in the United States [6]. To increase the recognition of PAs with advanced knowledge and experience in psychiatry, the NCCPA began offering a CAQ in psychiatry in May 2011 [7]. As of January 2019, the NCCPA website reports that over 275 PAs have obtained the Psychiatry CAQ [8]. It is not known if the psychiatric CAQ has impacted PA utilization or autonomy.

NCCPA reports that 40.5% of all PAs are employed in the hospital, yet only 18.1% of psychiatric PAs are employed in the hospital [3]. This underutilization of PAs in the inpatient setting is unlike other medical setting, such as, surgery, trauma and inpatient medicine that has seen improved patient outcomes and decreased length of stay with PA utilization [9,10]. The purpose of this study is to increase the understanding of PA autonomy and supervision, reimbursement, structure of on-the-job training, requirement for CAQ specialization, and impact on physician workload in inpatient psychiatric care. For facilities interviewed that do not utilize PAs, this study seeks to understand the barriers to PA employment.

## Materials and methods

Study design

This qualitative study utilized semi-structured interviews that focused on PA autonomy and supervision, reimbursement, structure of on-the-job training, specialized certifications, and impact on physician workload in inpatient psychiatric care. For facilities not utilizing PAs, the semi-structured interview focused on barriers to PA employment. A qualitative study design was used to allow for rich descriptions of these complex topics. The study was deemed exempt from IRB oversight by the University of Utah Institutional Review Board.

Participant selection

Participant selection included all inpatient psychiatric facilities in Utah, regardless of University-affiliation due to the interest of the authors in understanding state norms. Participant selection also included University-affiliated psychiatric facilities in the western half of the United States (Washington, California, Oregon, Texas, Nevada, New Mexico, Colorado, Arizona, Wyoming, Idaho, Montana, Nebraska, Oklahoma and Kansas). Participants interviewed were administrators at their respected facilities. Two approaches were taken to recruit participants for interviews: internet search and the snowball method. An internet search yielded a list of University-affiliated hospitals in the United States. That list was used to identify facilities and email addresses for administrators who oversee inpatient psychiatric care. Additionally, participants were recruited using the snowball method, in which interviewees were asked for any additional contacts for inpatient psychiatric units potentially using PAs. Participant selection was deemed complete when data saturation was identified.

Data collection

We conducted semi-structured interviews over the phone. An interview guide was developed and used that had overarching, open-ended questions and specific follow-up questions to ask if not already answered by the interviewee. A pilot interview was conducted with a local psychiatric facility in order to assess the content of the interview guide and the length of the interview. No changes were considered necessary following the pilot interview. There were two interview guide templates used: (1) facility currently has PAs employed, and (2) never employed PAs. Refer to Appendix 1. For each interview, data were collected on PA autonomy and supervision, reimbursement, specialized certifications, structure of training, and impact on physician workload. All interviewers kept notes during the interview to capture responses.

Data analysis

Notes from all nine interviews were compiled. An inductive approach was used and constant comparison analysis was undertaken to identify themes in the data [9]. Themes emerged in each of the topic areas and were grouped with results provided for facilities utilizing PAs and facilities not utilizing PAs.

## Results

Forty facilities were contacted based on our participant selection, of which nine inpatient psychiatric facilities interviewed with a 22.5% response rate. Four facilities were currently utilizing PAs, and five had never utilized PAs. Refer to Table 1. Of the facilities studied, five were in Utah, three in Texas and one in Arizona. 44% of the facilities are University-affiliated, and 55% of them have 50 or fewer beds. Of the facilities utilizing PAs, there was a range of one to three PAs employed.

**Table 1:** Demographics of inpatient psychiatric facilities PA, physician assistant.

	Utilizing PAs	Number of PAs	Location	University-affiliated	Number of beds
Location A	yes	2	Utah	no	>100
Location B	yes	1	Utah	no	<50
Location C	yes	1	Texas	yes	<50
Location D	yes	1	Texas	yes	>100
Location E	no	0	Utah	no	<50
Location F	no	0	Utah	no	<50
Location G	no	0	Utah	no	<50
Location H	no	0	Arizona	yes	>100
Location I	no	0	Texas	yes	>100

Autonomy and supervision

**Figure 1:**
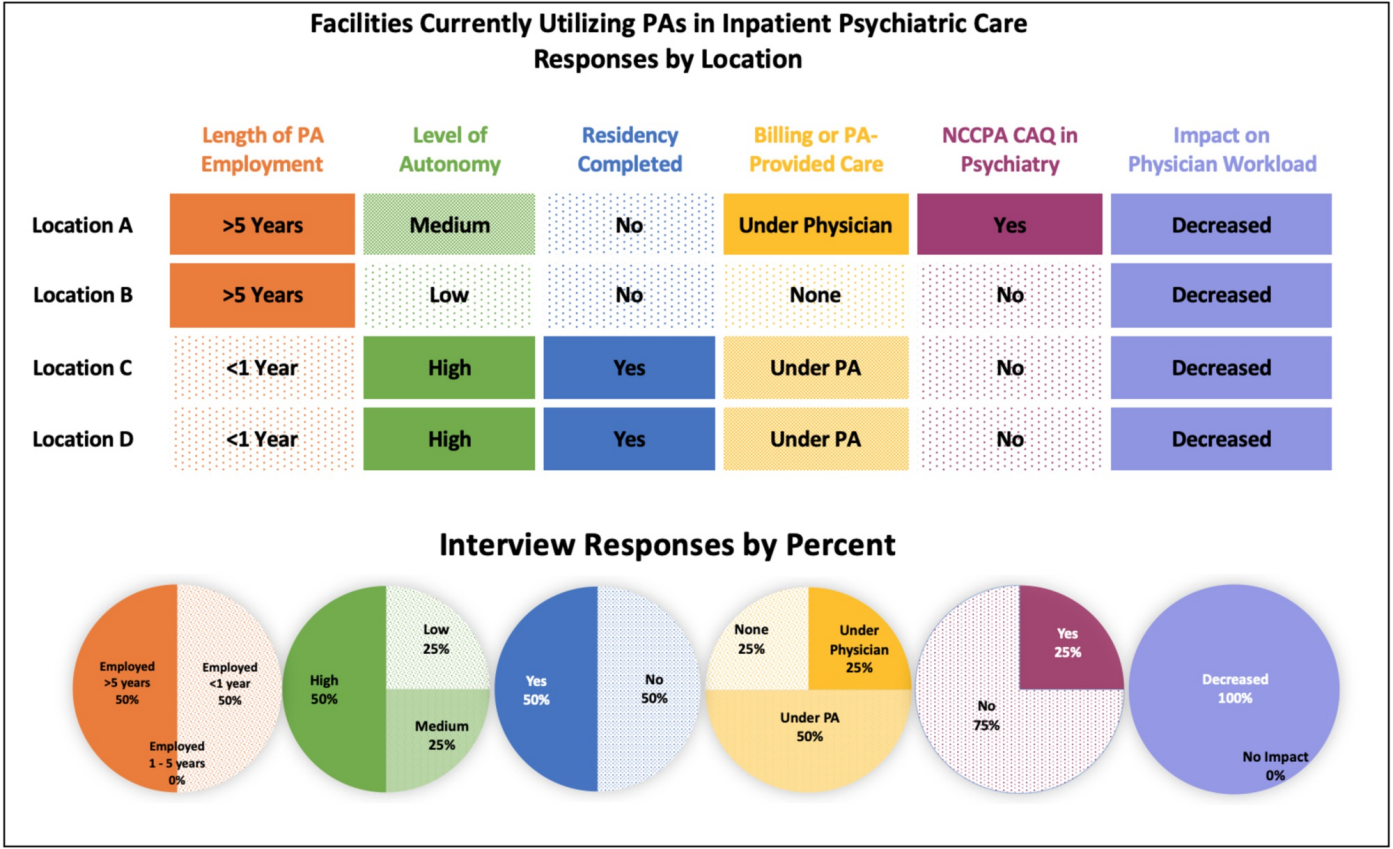
Facilities currently utilizing PAs in inpatient psychiatric care, responses by location PA, physician assistant; NCCPA, National Commission on Certification of Physician Assistant; CAQ, Certification of Advanced Qualification.

The themes regarding autonomy were categorized into high, moderate, or low autonomy. High autonomy was defined by the researchers as: PAs provide care for admitted patients independently without a requirement that the supervising physician see the patient. Moderate autonomy was defined as: PAs provide care, but the physician is required to see the patient at some point during care. Low autonomy was defined as: PAs see the patient but physician manages care. PAs at 40% of the locations were described as having a high level of autonomy, 40% as moderate, and 20% as low. Refer to Figure 1. Wide variation in autonomy is noted. For example, at Location B, the PAs only perform patient intake and discharge but do not provide care while patients are in the hospital. While at Location A, the PAs provide all the patient care during hospitalization, but physicians are required to see the patient at discharge and supervision consists of chart review. Location A stated the following regarding autonomy: “The PAs have been here virtually since the beginning and do everything that doctors do.” At Locations C and D, chart review is all that is required for the PA-provided care, including on the day of discharge.

Reimbursement

Variations in billing were seen from facility to facility. Refer to Figure 1. Location A bills under the supervising physician with only chart review. Location B allows a PA to complete the history and physical exam for patient intake, and they do not bill for the PA-provided care. After the physician takes over care, billing is performed under the physician. Locations C and D reported that they bill under the PA unless the attending had seen the patient that day, in which case billing is under the physician.

Training structure

Half of the facilities did not verbalize a preference for postgraduate residency training prior to employment. However, locations C and D preferred to hire PAs who had specifically completed a postgraduate residency in psychiatry. Refer to Figure 1. Locations A and B reported employing the same PAs for many years, and did not have a well-defined training structure. Location B noted that at the time of hiring, PAs will shadow physicians and nurse practitioners (NPs) for a variable length of time depending on previous experience. Locations C and D reported training new PA hires by shadowing and working closely with a PA who has completed a psychiatric residency.

Specialized certifications

The NCCPA CAQ in psychiatry was required by one facility prior to employment while the other three facilities did not require specialized certification. Refer to Figure 1. Locations C and D did not require the NCCPA CAQ in psychiatry but stated that they highly preferred it, and Location D was considering making it a future requirement. Location A billed under its PAs and required them to have the NCCPA CAQ in psychiatry, as it was determined to be a requirement by certain insurance companies for billing. Location B did not require the NCCPA CAQ, although its employed PA did have the certification.

Impact on physician workload

All the facilities reported that PAs reduced the physicians’ workload (Figure 1). Additionally, all the locations reported high satisfaction with the PAs. Location D stated, “We love our PAs,” and Location C stated, “They are so helpful.”

Facilities not utilizing PAs

**Figure 2:**
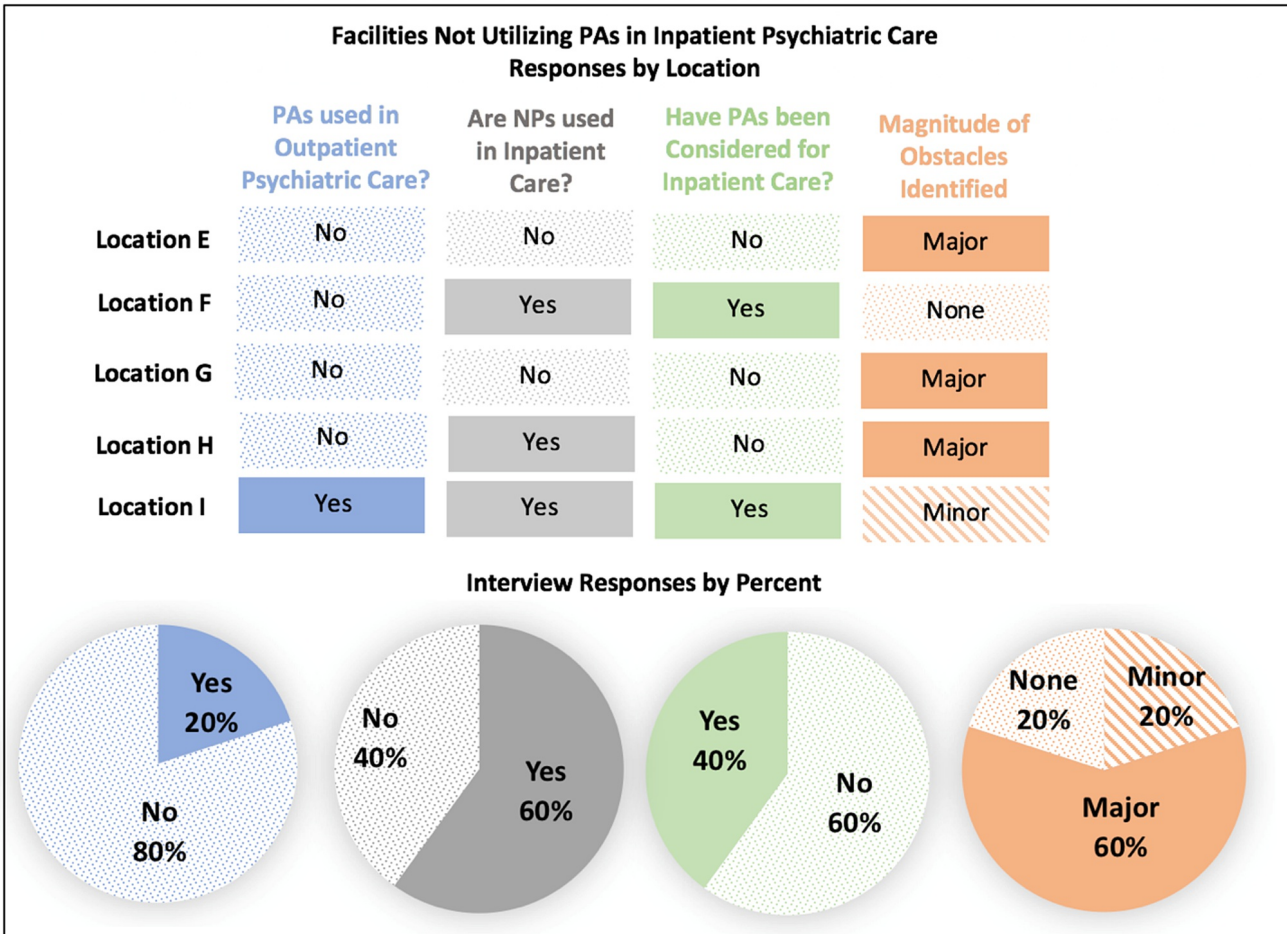
Facilities not utilizing PAs in inpatient psychiatric care, responses by location NP, nurse practitioner; PA, physician assistant.

At the five facilities interviewed, 60% of the facilities had not considered utilizing PAs. The other 40% had considered them but preferred physicians or NPs and currently utilize NPs in their inpatient unit. Refer to Figure 2. Interestingly, 60% of the facilities are utilizing NPs for inpatient care. In addition, 80%of the facilities also reported that they do not utilize PAs in outpatient care. Barriers to utilizing PAs were categorized into major, minor, or no barriers. Sixty percent of the facilities reported major barriers to hiring PAs, which the researchers defined as major barriers billing constraints, legal constraints, and hospital regulations precluding the use of PAs. Twenty percent of the facilities reported minor barriers which were defined to include historic preference and perceptions of the overall level of PA training. Principal themes across all facilities for not hiring PAs were as follows: historic preference for physicians or NPs; concerns or constraints imposed by the hospital about supervision and billing; belief that PAs have inadequate training for inpatient psychiatric care. Location E stated, “we have not thought of them.” Location F reported that it has interviewed PAs in the past and thus sees no barriers to hiring them, stating, “we would hire PAs but do not have any positions open at this time."

## Discussion

Less than half of the facilities were found to utilize PAs with multiple autonomy and reimbursement models. Due to differences in autonomy, the overall utilization of PAs appears to vary greatly between these locations. For example, some inpatient psychiatric PAs provide all patient care and others only perform the admission and discharge exams. This variation was also noted in reimbursement for care, as some psychiatric inpatient PAs billed for their care and others did not. This shows that due to the infrequent use of PAs in inpatient psychiatric care, there is a lack of precedence for PA-billed care. Difference in PA utilization in inpatient psychiatry could be due in part to difference in state scope of practice (SOP) laws. This study interviewed facilities utilizing PAs in Texas and Utah. Both states have limitations in the number of PAs an MD may supervise, and Utah also has a requirement for chart-cosignature determined at the practice site [11]. These SOP limitations are not thought to limit inpatient PA utilization but likely difference in use stem from hospital regulations [12]. PAs and NPs have proven to be safe and cost-effective physician extenders in surgery, trauma, and inpatient internal medicine and therefore can appropriately bill for these services [9,10]. Inpatient psychiatric PA practice should mirror these other specialties. To support this, further research should focus on the quality and cost-effectiveness of PAs in inpatient psychiatry.

Most inpatient psychiatric facilities are not requiring psychiatric residencies nor NCCPA CAQ certifications, but upon hire, no well-defined training structure is seen. Although PAs clearly have the potential to contribute positively to the discipline, there are inconsistencies in training that need to be addressed. As expected, training was found to be facility-dependent and had a broad span in training time. The belief is that more residency programs could ease the burden of training on individual facilities and open up avenues for more PAs to work in inpatient psychiatric care [5]. However, with only three active post-graduate psychiatric residency programs there are limited opportunities to strengthen the PA psychiatric workforce. Future research needs to examine the impact of both PA residencies and NCCPA CAQ on PA employment in psychiatric settings.

All facilities utilizing PAs reported a decreased workload for physicians and satisfaction with their work. This is the first study of its kind assessing psychiatrist workload and satisfaction. The perceived benefit of PAs to the health care team has also been noted by a survey of surgical residents [13]. A study of family medicine found that having a PA increased the family practice physician panel by 410 patients [14]. Further research is needed to quantify the impact that PAs have in psychiatric care both in patient access to care and patient outcomes. The majority of facilities surveyed are not using PAs and cited major barriers to PA utilization. Surprisingly, only one of these facilities is utilizing NPs. With the projected shortage of psychiatrists, it is unlikely that half of the facilities interviewed can continue being staffed solely by physicians. Major barriers included legal constraints and hospital regulations, which support the need for increased awareness of PA versatility, capability, and training. Some facilities reported being unaware that PAs are qualified to work in inpatient psychiatric care. This study highlights the need for policymakers and PA advocates to educate hospital administrators and oversight committees regarding PA qualifications and their scope of practice in psychiatry.

Study limitations

Although this study contributed to a minimal body of research on this topic, there are several limitations. This study involved only nine facilities from three states, which limits generalizability. Also, due to wide variations in state laws, the findings from this study regarding supervision and reimbursement may or may not be applicable to other locations. This study relied on responses from the administrator at each facility, which may not be representative of the entire facility and could thus be biased by personal experience with PAs. To address this, future research could include multiple interviews at the same location. This study is a qualitative analysis that does not provide a quantitative analysis of psychiatric PA utilization and trends. Lastly, all interviewers were involved in PA training, so interviewer bias could have influenced interviewee responses.

## Conclusions

The demand for psychiatric care is increasing while there is a national shortage of psychiatric care providers. The majority of the facilities did not utilize PAs and either preferred physicians or were unaware of the qualifications of PAs. Of the facilities utilizing PAs, there is wide variation in PA utilization and reimbursement models in psychiatric inpatient care. Yet, of those same facilities, there is a high level of satisfaction, reinforcing that PAs can provide high-quality care in inpatient psychiatric settings. Future research is needed to quantify the impact of PAs in psychiatry on physician workload, patient access to care and patient outcomes. Increased knowledge of PA qualification and PA scope of practice in psychiatry is needed to decrease barriers to PA employment in inpatient psychiatry.

## Appendices

Appendix 1- Inpatient Psychiatry Questionnaire

Introduction:

Hello Mr/Mrs.__________,

I am a PA student at the University of Utah working on a research project with the University of Utah. Would you be willing to answer a few questions about Physician Assistants in inpatient psychiatry? This should take no more than 10 minutes of your time.

Have you ever had Physician Assistants working in inpatient psychiatric care at your facility?

If Yes, ask:     Are PAs currently working there?                             

If Yes - Use Template 1          If No- Use Template 2

Template 1 - PAs Currently Employed.

General Background Information

1.     How many PAs do you have working on staff in in-patient psych?

2. How long have you had PAs on staff?

PA Autonomy and Reimbursement

3. How are the PAs being utilized - what is their scope and role in patient care?

Supplemental questions:

What is the supervision structure? (i.e. Do physicians see all the PA’s patients? Do they just see them at intake and discharge? Do they just do chart review?)

Are your PAs working at the full scope of practice permitted by state laws, or does the hospital administration have different scope of practice guidelines?

4. How do you bill for PA-provided care?

Supplemental questions:

Does a Physician have to see every patient every day for billing purposes?

Is the care billed for under the PA or the MD?

Structure of on-the-job training

5. Can you describe the training model you use for PAs?         

Supplemental questions:

How effective do you think your training model is?

            How long (# of months) does training last?

            Are you contemplating any changes to it?

Specialized Certification

6. Does your facility require your PAs to have any special certifications (for example, NCCPA’s CAQ in psychiatry)?

Supplemental Questions:

Are any pursuing it now? Do you offer support or training in gaining this?

General Concluding Information:

7. How satisfied are you with having PAs as part of the practice?

8. Have you found that having PAs in the practice have negatively/positively impacted the workload of the physicians?

9. Do you know of other facilities that are using PAs in in-patient psych? Can you give us contacts there?

Template 2 - PAs Employed in the past, not currently there.

General Background Information

Why aren’t you currently utilizing PAs?

2.     How many PAs did you have working on staff in in-patient psych?

3. How long did you have PAs on staff?

PA Autonomy and Reimbursement

4. How were the PAs being utilized - what was their scope and role in patient care?

Supplemental questions:

What was the supervision structure? (i.e. Did physicians see all the PA’s patients? Did they just see them at intake and discharge? Did they just do chart review?)

Did your PAs working at the full scope of practice permitted by state laws, or did the hospital administration have different scope of practice guidelines?

5. How did you bill for PA-provided care?

Supplemental questions:

Did a Physician have to see every patient every day for billing purposes?

Was the care billed for under the PA or the MD?

Structure of on-the-job training

5. Can you describe the training model you used for PAs?      

Supplemental questions:

How effective do you think your training model was?

            How long (# of months) did training last?

Specialized Certification

7. Did your facility require your PAs to have any special certifications (for example, NCCPA’s CAQ in psychiatry)?

Supplemental Questions:

Did you offer support or training in gaining this?

General Concluding Information:

8. How satisfied were you with having PAs as part of the practice?

9. Did you find that having PAs in the practice negatively/positively impacted the workload of the physicians?

10. Do you know of other facilities that are using PAs in in-patient psych? Can you give us contacts there?
